# Different doses of supplemental vitamin D maintain interleukin-5 without altering skeletal muscle strength: a randomized, double-blind, placebo-controlled study in vitamin D sufficient adults

**DOI:** 10.1186/1743-7075-9-16

**Published:** 2012-03-09

**Authors:** Tyler Barker, Thomas B Martins, Harry R Hill, Carl R Kjeldsberg, Vanessa T Henriksen, Brian M Dixon, Erik D Schneider, Adam Dern, Lindell K Weaver

**Affiliations:** 1 The Orthopedic Specialty Hospital, Murray, UT, 84107, USA; 2 ARUP Laboratories, Institute for Clinical and Experimental Pathology, Salt Lake City, UT, 84108, USA; 3 Department of Pathology, University of Utah, Salt Lake City, UT, 84132, USA; 4 USANA Health Sciences, Inc., Salt Lake City, UT, 84120, USA; 5 Hyperbaric Medicine, Intermountain Medical Center, Murray, UT, 84107, USA; 6 LDS Hospital, Salt Lake City, UT, 84143, USA; 7 University of Utah, School of Medicine, Salt Lake City, UT, 84132, USA; 8 The Orthopedic Specialty Hospital, 5848 S. Fashion Blvd., Murray, UT, 84107, USA

**Keywords:** Vitamin D, Interleukin-5, Skeletal muscle function

## Abstract

**Background:**

Supplemental vitamin D modulates inflammatory cytokines and skeletal muscle function, but results are inconsistent. It is unknown if these inconsistencies are dependent on the supplemental dose of vitamin D. Therefore, the purpose of this study was to identify the influence of different doses of supplemental vitamin D on inflammatory cytokines and muscular strength in young adults.

**Methods:**

Men (*n *= 15) and women (*n *= 15) received a daily placebo or vitamin D supplement (200 or 4000 IU) for 28-d during the winter. Serum 25-hydroxyvitamin D (25(OH)D), cytokine concentrations and muscular (leg) strength measurements were performed prior to and during supplementation. Statistical significance of data were assessed with a two-way (time, treatment) analysis of variance (ANOVA) with repeated measures, followed by a Tukey's Honestly Significant Difference to test multiple pairwise comparisons.

**Results:**

Upon enrollment, 63% of the subjects were vitamin D sufficient (serum 25(OH)D ≥ 30 ng/ml). Serum 25(OH)D and interleukin (IL)-5 decreased (*P *< 0.05) across time in the placebo group. Supplemental vitamin D at 200 IU maintained serum 25(OH)D concentrations and increased IL-5 (*P *< 0.05). Supplemental vitamin D at 4000 IU increased (*P *< 0.05) serum 25(OH)D without altering IL-5 concentrations. Although serum 25(OH)D concentrations correlated (*P *< 0.05) with muscle strength, muscle strength was not changed by supplemental vitamin D.

**Conclusion:**

In young adults who were vitamin D sufficient prior to supplementation, we conclude that a low-daily dose of supplemental vitamin D prevents serum 25(OH)D and IL-5 concentration decreases, and that muscular strength does not parallel the 25(OH)D increase induced by a high-daily dose of supplemental vitamin D. Considering that IL-5 protects against viruses and bacterial infections, these findings could have a broad physiological importance regarding the ability of vitamin D sufficiency to mediate the immune systems protection against infection.

## Introduction

Vitamin D status is influenced by a variety of factors, including but not limited to geographical latitude, season and supplement use. Living in extreme northern and southern climates and the winter season result in low vitamin D levels [1,2], while supplemental vitamin D increases serum 25-hydroxyvitamin D (25(OH)D) in a dose-dependent fashion [3-5]. Serum 25(OH)D is the best indicator of vitamin D status [6], but 1α,25-dihydroxyvitamin D_3 _(1,25(OH)D) is the active form of vitamin D that regulates biological functions. In kidney and immune cells, 1α-hydroxylase (1α-OHase) converts 25(OH)D to 1,25(OH)D. This enzymatic conversion is limited by substrate availability since 1α-OHase operates below its *K*_m_. Thus, an adequate circulating 25(OH)D concentration is necessary to maintain substrate availability for the enzymatic conversion of vitamin D to its active form.

Low serum 25(OH)D concentrations result in skeletal muscle weakness [7,8], and in pathophysiological and physiological conditions, correlate with muscular strength or physical performance [9-14]. These findings suggest that increasing serum 25(OH)D concentration improves muscular strength. Consistent with this deductive reasoning, supplemental vitamin D reversed vitamin D deficiency-induced weakness in elderly [10,15] and in patients with diverse diseases or illnesses [16-18]. Although we previously demonstrated that serum 25(OH)D concentration is influential on muscle strength recovery following ligamentous injury and surgery in active adults [19], it is unknown if supplemental vitamin D increases muscular strength simultaneous with serum 25(OH)D concentrations in young, reportedly active and healthy adults.

Vitamin D regulates inflammatory cytokines, which orchestrate host defenses by the immune system. Interferon (IFN)-γ is a cytokine that regulates T helper 1 (T_H1_)-type cells and pathways [20-22], and in immune cells, is regulated by vitamin D [23-25]. Interleukin (IL)-5 and IL-10 regulate T_H2_-type cell pathways [26-30] and are modulated by vitamin D [31-34]. Despite previous reports identifying the influence of vitamin D on cytokine production *in vitro*, evidence illustrating concurrent alterations in serum 25(OH)D and cytokine concentrations in humans is more ambiguous. For example, in healthy-adults during the winter, low serum 25(OH)D concentrations corresponded with increased IFN-γ and IL-10 production [35], while supplemental vitamin D (cholecalciferol) increased IL-10 in congestive heart failure patients [36] but was ineffective at modulating IFN-γ, IL-5 or IL-10 in overweight or obese subjects [37]. Currently, it is unknown if supplemental vitamin D modulates inflammatory cytokines concomitantly with serum 25(OH)D concentrations in young, healthy adults during the winter.

To date, one study has examined the influence of different doses of supplemental vitamin D on serum 25(OH)D simultaneously with circulating cytokines in healthy adults during the winter, and the range of supplemental vitamin D (cholecalciferol) doses were narrow (i.e., 200, 400 and 600 IU/d) [38]. Additionally, it is unknown whether short-term and different doses of supplemental vitamin D modulate muscular strength similarly despite divergent serum 25(OH)D concentration responses. Therefore, the purpose of this study was to identify the influence of low and high doses of supplemental vitamin D on serum 25(OH)D, inflammatory cytokines and muscular strength in young adults during the winter. We hypothesized that a 'low' daily dose of supplemental vitamin D maintains serum 25(OH)D concentration, and that a 'high' daily dose is necessary to modulate circulating cytokines and muscular strength in young adults during the winter. To test this hypothesis, subjects were provided a daily placebo supplement or supplemental vitamin D at different doses (200 or 4000 IU) in a randomized, double-blind experimental design. Supplements were taken for 28-d during the winter, and serum 25(OH)D, cytokine and muscular strength measurements were performed prior to and during the supplemental intervention in young, reportedly active and healthy adults. We demonstrate in young adults who were vitamin D sufficient prior to supplementation that a low-daily dose of supplemental vitamin D prevents serum 25(OH)D and IL-5 concentration decreases, and that muscular strength does not parallel the 25(OH)D increase induced by a high-daily dose of supplemental vitamin D.

## Methods

The Institutional Review Board at Intermountain Healthcare (Murray, UT, USA) approved this study. Subjects were informed of the experimental protocol and procedures and provided both written and verbal consent prior to participation. Recreationally active (minimum of 30 minutes of continuous exercise at least 3 time per week for 1 year prior to enrollment), non-smoking males and females between the ages of 18 and 45 years of age were recruited to participate in this study. Subjects were excluded from participation if they experienced a lower-extremity injury or injuries during the year prior to enrollment. Subjects were also excluded from participation if they were taking a daily dietary-supplement during the previous year, planning on increasing or decreasing the amount of time spent in the sun or tanning bed, or traveling south of 37°N latitude during enrollment. Subjects were asked to refrain from using any other supplements during study participation. Data was collected during two successive winters. First, between December 1, 2009 and April 26, 2010, and second, between December 1, 2010 and March 15, 2011 in Salt Lake City, UT USA (40°N latitude). We chose to collect data during the winter because that is when vitamin D levels are at their nadir. This occurs because the solar angle during the winter is inadequate to stimulate cutaneous vitamin D synthesis from sun (ultraviolet B radiation) exposure.

### Study design and protocol

This study consisted of a randomized, double-blind, placebo-controlled experimental design. Subjects were randomly assigned to one of three groups: vitamin D (cholecalciferol) supplementation at: (1) 200 or (2) 4000 IU, or (3) a matching-placebo. Supplements were taken daily for 28-d. Supplements were permutated into random blocks of six. Subjects were asked to refrain from using aspirin, ibuprofen, naproxen sodium, and acetaminophen throughout the duration of the study.

It should be noted, that upon study initiation, 200 IU/d of vitamin D was the recommended-adequate intake and 4000 IU/d was double the upper limit for the targeted population. Currently, 200 IU/d is lower than the current recommended dietary allowance and 4000 IU is the upper limit for the studied population. These recommendations are provided by the Institute of Medicine for the United States and Canada [39]. Although recommendations changed during the course of this study, the experimental design (randomized, double-blind, placebo-controlled) and the examination of two different doses of supplemental vitamin D are strengths of this investigation.

### Analytical procedures

Each subject provided five fasting blood draws: (1) prior to (Pre), and (2) 7-, (3) 14-, (4) 21- and (5) 28-d after supplementation. Subjects were asked to refrain from physical activity 72-h prior to each blood draw to ensure that prior activity did not confound serum 25(OH)D and cytokine concentration results. Fasting blood draw samples were obtained from the antecubital vein into one 4.0 ml purple-top Becton Dickinson (BD) Vacuatainer tube (K2 EDTA 7.2 mg), one 4.5 ml light green-top BD Vacutainer tube (PST Gel and Lithium Heparin, 83 units), and one 6.0 ml red-top serum BD Vacutainer tube. Plasma was separated by centrifugation (Heraeus Labofuge 400 series, Buckinghamshire, England) at 2400 *g *for 6 min within 20 min of sample collection. Following separation, plasma samples were sent to clinical laboratories for analytical chemistries (see below). Serum was separated by centrifugation (VWR International, Clinical 50 Centrifuge) at 1100 *g *for 20 min within 20 min of sample collection and after coagulation. Serum samples were immediately stored at -80°C (Revco Freezer, GC Laboratory Equipment, Asheville, NC, USA) until the day of 25(OH)D and cytokine concentration analyses (see below).

#### Serum 25(OH)D concentrations

Serum 25(OH)D concentrations (ng/ml) were measured at USANA Health Sciences, Inc. (Salt Lake City, UT USA) using a modified Bligh-Dyer technique for extraction [40] and a scaled-down and modified method as previously described [41]. Briefly, 100 μl of serum was added to 400 μL of a 2:1 methanol:chloroform solution containing dueterated 25(OH)D_3 _as an internal standard (10 ng/mL in the stock solution; 40 ng/mL final concentration) in a 2 mL centrifuge tube. Samples were immediately vortexed and allowed to sit on ice for 10 min. Samples were then spun at 15,000 *g *for 5 min and the supernatant transferred to a new 2 mL centrifuge tube containing 500 μL chloroform. Samples were vortexed and allowed to stand for 5 min. To achieve phase separation, 750 μL of ddH_2_O was added, vortexed, and centrifuged for 2 min at 15,000 *g*. The aqueous phase (top), and any debris between the two phases, was removed and discarded. The remaining organic phase was dried down to completeness in a centrifugal vacuum concentrator for 18 min at 45°C under negative pressure. The pellet was then resuspended in 100 μL of methanol and added to high performance-liquid chromatography (HPLC) vials.

Analytes were separated by injecting 10 μL into an Agilent HPLC (series 6410, Model G6410B, Santa Clara, CA USA) and a Phenomenex Inertsil 3 micron, 150 × 4.60 mm column. Method conditions were: 0-8 min, 90% MeOH/10% (0.03% formic acid in water); 8-15 min, 30% MeOH/70% 2-propanol; 15-20 min, 90% MeOH/10% (0.03% formic acid in water). 25(OH)D_2 _and 25(OH)D_3 _were detected on an Agilent tandem mass spectrometer (Series 6410, Model G6410B, Sant clara CA USA) using atmospheric pressure chemical ionization (APCI) detection (350°C gas temperature, 400°C vaporizer). The 25(OH)D_3_, dueterated 25(OH)D_3 _and 25(OH)D_2 _precursor ions were 383.3, 386.3 and 395.4, respectively. The 25(OH)D_3_, dueterated 25(OH)D_3 _and 25(OH)D_2 _productions were 365.3, 368.3 and 208.9, respectively. Serum 25(OH)D_2 _and 25(OH)D_3 _concentrations were determined relative to authentic standards and corrected for recovery of the 25(OH)D_3 _internal standard. The detection limit was determined to be < 1 ng/ml for all analytes. The sum of 25(OH)D_2 _and 25(OH)D_3 _concentrations was used as the 25(OH)D total concentration. However, since serum 25(OH)D_2 _was not detected in any subjects, serum 25(OH)D total concentrations are referred to as serum 25(OH)D concentrations hereafter.

#### Cytokines

A multiplex microsphere-bead array was used to measure a number of circulating inflammatory cytokines (pg/ml) as described previously [42,43]. Briefly, IFN-γ, IL-5 and IL-10 were quantitated using a multiplexed sandwich capture assay developed in the ARUP Institute for Clinical and Experimental Pathology (University of Utah, Salt Lake City, UT, USA) using the Luminex Multi-Analyte Profiling system (Luminex, Austin, TX, USA) [42].

We have reported the procedural precision of this multiplex-cytokine bead assay previously [42,43]. In brief, using independent control samples of low, medium, and high concentrations the coefficient of variation was calculated on 5 replicates. The intra-assay coefficient of variation was predominantly less than 10%. The inter-assay coefficient of variation was predominantly less than 15% for the medium and high control samples and up to ~30% for the low control sample [42,43].

#### Clinical chemistries

Plasma parathyroid hormone (PTH; pg/ml) and calcium (mg/dl) concentration measures were performed at ARUP Laboratories (Salt Lake City, UT USA). Plasma creatinine (mg/dl) concentrations were measured at the Central Laboratory with Intermountain Healthcare (Murray, UT USA).

To assess renal function prior to and following supplementation, the estimated glomerular filtration rate (eGFR; ml/min/1.73 m^2^) was calculated by the following equation:

$$\text{eGFR}=186\times {\left(\text{creatinine}/88.4\right)}^{-1.154}\times {\left(\text{age}\right)}^{-0.203}\times \left(0.742\;\text{if}\;\text{female}\right)\times \left(1.210\;\text{if}\;\text{black}\right)$$

#### Single-leg strength testing

Subjects with strength or power output asymmetry (i.e., > 5% difference in peak isometric or power output between legs) were excluded from participation. Leg strength from a single randomly selected leg was performed prior to (Pre) and 28-d after (Post) placebo or vitamin D supplementation. Single-leg strength testing was performed on a horizontal plyo-press (Athletic Republic, Park City, UT USA). Plyo-press output data were measured from output signals obtained from a mounted force plate (Advanced mechanical Technology, Watertown, MA USA) and displacement transducer (UniMeasure PA-50-NJC, Corvallis, OR USA). All data were sampled at 200 Hz with a low-pass filter at 10 Hz using DartPower software (Athletic Republic, Park City, UT USA, version 2.0). Before every testing session, the mounted force plate was zeroed and load calibrated.

For the single-leg peak isometric force measurements, the plyo-press sled was adjusted for each subject to align the knee and hip joint flexion angles to 90° with the abdominal, lower back region secured and stabilized to the plyo-press sled with a harness. The plyo-press sled position was documented and reproduced to achieve the desired knee and hip joint flexion angles at the follow-up visits. Leg selection was randomized. Subjects were verbally instructed and strongly encouraged to exert maximal force against the mounted force platform. Each subject performed three maximal single-leg isometric contractions. Each isometric contraction was 3 sec in duration and separated by approximately 1 min. Peak isometric force (N/kg) was defined as the highest resultant force applied during the 3 sec test for each trial. To calculate average isometric force, peak isometric forces from the 3 trials at each visit were averaged.

Single-leg peak power output measures followed the single-leg isometric contractions and were measured on the same horizontal plyo-press with the same securing procedures described above. Starting from an extended position (full extension = 0°), subjects performed repetitive single-leg jumps (i.e., hip and knee flexion-extension cycles) as fast as possible. Subjects were instructed to jump as high as possible. Each test was 20 sec in duration with the weight-stack resistance set at 75% of body mass. The time-aligned product of the resultant forces (N) acquired from the force platform and weight-stack velocities (m/s) data obtained from the displacement transducer were used to calculate power output and expressed relative to body mass (W/kg). Peak power output was defined as the highest power output produced during the 20 sec test for each leg. The peak power output for each jump during the 20 sec test were averaged to provide an average power output for each testing session in each leg.

### Statistical analyses

To achieve normality, rank transformations were performed on eGFR, serum 25(OH)D changes from Pre, and serum cytokine changes from Pre. Transformations were checked for normality with a Kolmogorov-Smirnov test. Statistical significance of data were assessed with a two-way (time, treatment) analysis of variance (ANOVA) with repeated measures, followed by a Tukey's Honestly Significant Difference to test multiple pairwise comparisons. One-way ANOVA tests were performed on subject characteristics. Relationships between variables were examined with a Pearson Product Moment Correlation. All statistical analyses were performed with SysStat software (SigmaPlot 10.0, SigmaStat 3.5, Chicago, IL, USA). Statistical significance was set at a *P *< 0.05. Data presented as mean (SD) unless otherwise noted.

## Results

### Subject characteristics

Subject characteristics were similar between groups (Table 1). For example, there was less than a 6% difference in body mass, and importantly, less than a 1% difference in BMI between vitamin D groups (i.e., 200 vs. 4000 IU; Table 1). This is a strength of the present study because it identifies similar body sizes between supplemental vitamin D groups.

**Table 1:** Subject characteristics

	Placebo	200 IU	4000 IU
n (males/females)	10 (5/5)	10 (4/6)	10 (6/4)

Age (y)	30.2 (4.8)	26.6 (3.2)	29.0 (5.0)

Height (cm)	167 (14)	169 (9)	173 (9)

Body mass (kg)	72.5 (17.2)	69.4 (17.2)	73.3 (15.3)

Body mass index (kg/m^2^)	25.5 (3.0)	24.0 (4.1)	24.2 (3.6)

Data presented as mean (SD) unless otherwise noted

Twenty-nine subjects were Caucasian; one subject was African American (placebo group)

### Baseline vitamin D status

Upon enrollment, serum 25(OH)D concentrations were 32.2 (10.0) ng/ml. Prior to randomization, 13.3 (*n *= 4), 23.3 (*n *= 7) and 63.3% (*n *= 19) of the subjects were vitamin D deficient (serum 25(OH)D ≤ 20 ng/ml), insufficient (serum 25(OH)D 21-29 ng/ml) or sufficient (serum 25(OH)D ≥ 30 ng/ml), respectively.

Following randomization, 30, 20 and 50% of the subjects in the placebo group started study participation vitamin D deficient, insufficient or sufficient, respectively. In the 200 IU group, 10% were deficient, 20% were insufficient and 70% were sufficient, and in the 4000 IU group, 30% were insufficient and 70% were sufficient when starting study participation. Based on these data, starting vitamin D status was sufficient at the group level and in groups assigned to different supplemental treatments.

### Serum 25(OH)D concentrations

**Figure 1:**
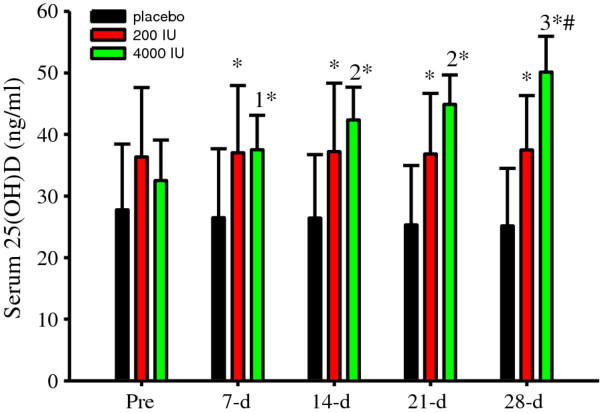
**Serum 25(OH)D concentrations (ng/ml) were significantly (*P *< 0.001, treatment, time interaction) modified following supplementation**. Although there were no significant differences in the placebo or 200 IU groups, serum 25(OH)D concentrations increased after 7-d (^1 ^*P *< 0.001 vs. Pre) and continued to increase thereafter (^2^*P *< 0.001 vs. Pre and 7-d; ^3^*P *< 0.001 vs. Pre, 7-d, 14-d and 21-d) in the 4000 IU group. Serum 25(OH)D concentration differences between groups at Pre were not significant. However, serum 25(OH)D concentrations were significantly (*P < 0.05 vs. placebo) increased in the 200 and 4000 IU groups compared to those in the placebo group after supplementation. By 28-d, serum 25(OH)D concentrations were significantly (^#^*P *< 0.05 vs. 200 IU) increased in the 4000 IU compared to those in the 200 IU group. Data presented as mean (SD).

Treatment efficacy was evaluated by measuring serum 25(OH)D concentrations. Serum 25(OH)D concentrations progressively and significantly (*P *< 0.05) increased in 4000 IU group and compared to those in the placebo and 200 IU groups (Figure 1). Surprisingly, supplemental vitamin D at 200 IU increased serum 25(OH)D concentrations compared to those in the placebo group (Figure 1).

**Figure 2:**
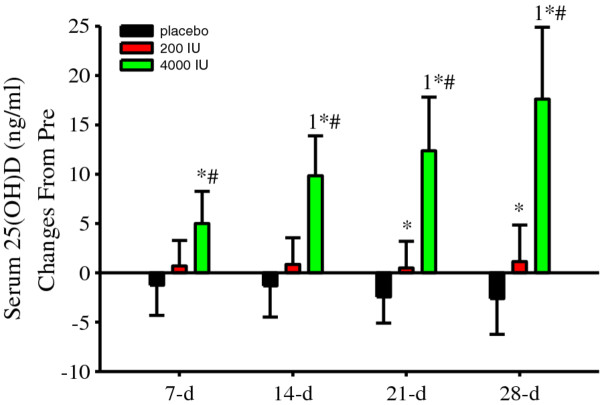
**Serum 25(OH)D concentration (ng/ml) changes from Pre were significantly (*P *< 0.005, treatment, time interaction) modulated following supplemental vitamin D**. Serum 25(OH)D changes from Pre were significant (^1^*P *< 0.05 vs. 7-d) in the 4000 IU group and compared to the corresponding placebo (*P < 0.05 vs. placebo) and 200 IU (^#^*P *< 0.05 vs. 200 IU) changes. Serum 25(OH)D concentration changes were also significantly (*P < 0.05 vs. placebo) different between the placebo and 200 IU groups at 21-d and 28-d. Data presented as mean (SD).

Since vitamin D status tended (*albeit *non-significantly) to be higher in the 200 IU group upon study enrollment (Figure 1), serum 25(OH)D was normalized to Pre concentrations (change from Pre). Serum 25(OH)D concentration changes were not significantly different in the placebo or 200 IU groups (Figure 2). In contrast, serum 25(OH)D concentration changes significantly (*P *< 0.05) increased in the 4000 IU group and compared to those in placebo and 200 IU groups (Figure 2). At 21-d and 28-d, serum 25(OH)D concentration changes were significantly (*P *< 0.05) different between the placebo and 200 IU groups (Figure 2).

Altogether, these data indicate that 200 IU of supplemental vitamin D protected against a seasonal decrease while 4000 IU induced a rapid increase in serum 25(OH)D concentrations.

### Clinical chemistries

Due the anticipated seasonal decrease (which could change vitamin D status from sufficient to insufficient) and the efficacy of supplemental vitamin D at 4000 IU to increase serum 25(OH)D concentrations, PTH and calcium were measured in each blood draw sample. PTH and calcium concentrations were not significantly different within or between groups (Table 2). Along with creatinine measures, the eGFR was calculated to assess renal function. Creatinine and the eGFR were not significantly different within or between groups (Table 2). Thus, the seasonal decrease and supplemental vitamin D-mediated increase in serum 25(OH)D concentrations did not manifest in PTH, calcium, creatinine or eGFR alterations.

**Table 2:** Plasma clinical chemistries

	placebo	200 IU	4000 IU
PTH (pg/ml)			

Pre	36.1 (13.9)	36.1 (14.6)	31.4 (10.3)

7-d	40.7 (16.5)	35.8 (16.4)	30.8 (11.4)

14-d	38.5 (11.0)	31.7 (13.1)	30.3 (7.9)

21-d	35.0 (13.5)	31.1 (11.0)	25.9 (8.3)

28-d	35.5 (12.6)	33.0 (17.9)	32.1 (11.4)

calcium (mg/dl)			

Pre	9.22 (0.27)	9.36 (0.26)	9.32 (0.22)

7-d	9.06 (0.16)	9.21 (0.38)	9.38 (0.26)

14-d	9.15 (0.24)	9.22 (0.27)	9.41 (0.21)

21-d	9.09 (0.31)	9.38 (0.29)	9.30 (0.28)

28-d	9.16 (0.24)	9.39 (0.35)	9.35 (0.23)

creatinine (mg/dl)			

Pre	0.93 (0.08)	0.91 (0.14)	0.98 (0.17)

7-d	0.92 (0.08)	0.97 (0.16)	0.98 (0.15)

14-d	0.94 (0.09)	0.92 (0.13)	0.97 (0.17)

21-d	0.92 (0.13)	0.92 (0.15)	0.98 (0.15)

28-d	0.94 (0.16)	0.91 (0.15)	1.01 (0.13)

eGFR (ml/min/1.73 m^2^)			

Pre	90.9 (15.6)	91.9 (12.6)	87.7 (11.3)

7-d	91.1 (12.3)	85.5 (15.0)	87.5 (10.2)

14-d	89.1 (13.0)	89.7 (9.2)	89.5 (12.5)

21-d	91.6 (8.0)	91.0 (13.2)	88.0 (12.5)

28-d	90.2 (12.1)	92.1 (14.3)	84.7 (9.3)

Data presented as mean (SD)

### Inflammatory cytokines

**Figure 3:**
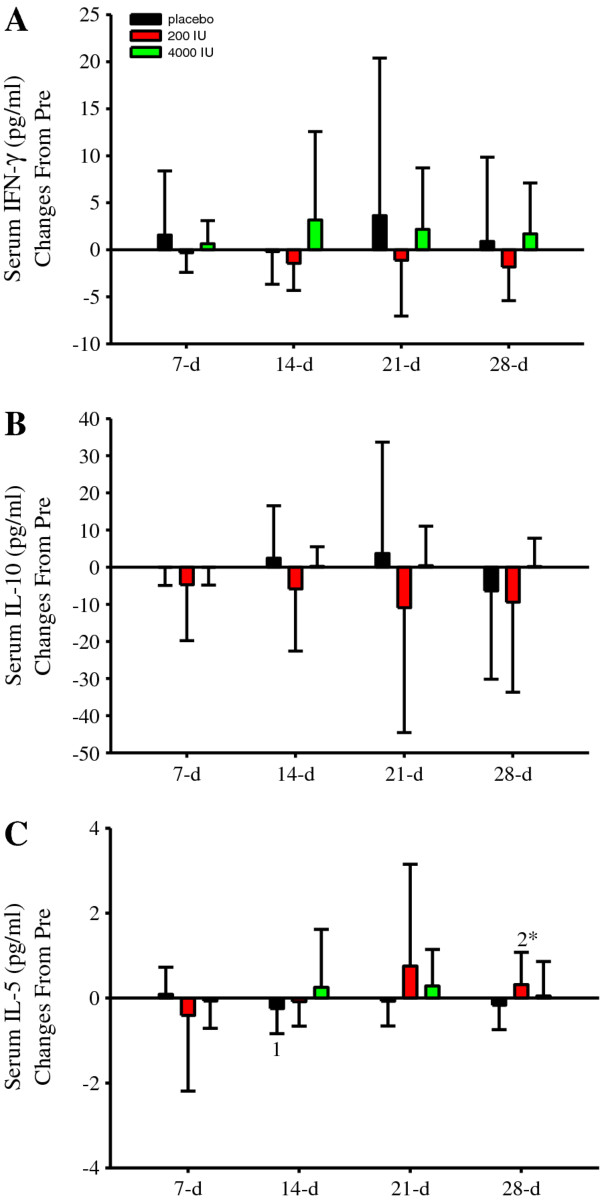
**Serum IFN-γ, IL-10 and IL-5 concentration (pg/ml) changes from Pre**. Serum IFN-γ (**A**) and IL-10 (**B**) concentration changes were not significantly different within or between groups. However, IL-5 (**C**) displayed a significant (*P *< 0.05) treatment, time interaction. Although no significant differences were observed within the 4000 IU group, there was a significant and transient decrease in IL-5 at 14-d (^1^*P *< 0.05 vs. 7-d) in the placebo and increase at 28-d (^2^*P *< 0.05 vs. 7-d and 14-d) in 200 IU group. At 28-d, IL-5 changes were also significantly (*P < 0.05) different between the placebo and 200 IU groups. Figure legend provided in '**A**'. Data presented as mean (SD).

Cytokine data was normalized to Pre concentrations because of variability. Following supplementation, IFN-γ (Figure 3A) and IL-10 (Figure 3B) were not significantly different. In contrast, there was a transient and significant (*P *< 0.05) IL-5 decrease in the placebo group (Figure 3C). Furthermore, IL-5 significantly (*P *< 0.05) increased in the 200 IU group and compared to that in the placebo group at 28-d (Figure 3C). Vitamin D supplementation at 4000 IU/d did not significantly change serum IL-5 concentrations (Figure 3C). When taken in the context that our cytokine data was variable and collected in healthy adults who were monitored for only 28-d during the winter without exposure to any undue distress, these robust and unique findings illustrate that supplemental vitamin D maintains serum 25(OH)D and IL-5 concentrations.

### Single-leg isometric forces and power outputs

**Figure 4:**
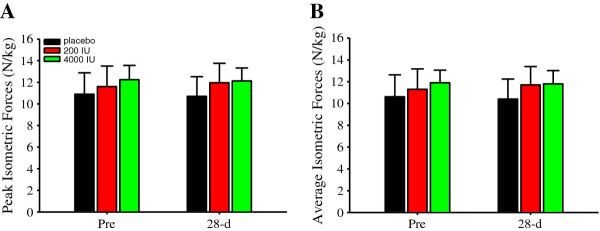
**Single-leg isometric forces (N/kg)**. Single-leg peak (**A**) and average (**B**) isometric forces were not significantly different within or between groups. Figure legend provided in '**A**'. Data presented as mean (SD).

**Figure 5:**
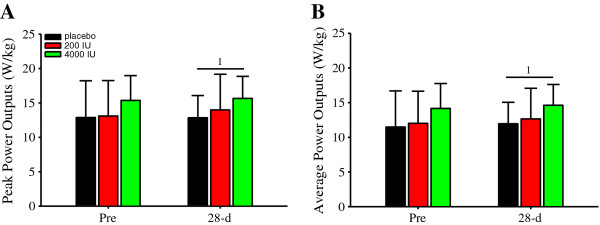
**Single-leg power outputs (W/kg)**. Single-leg peak (**A**) and average (**B**) displayed significant (both *P *< 0.05) main effects of time. Specifically, peak (**A**) and average (**B**) power outputs were significantly (^1^*P *< 0.05) greater at 28-d vs. Pre. Data presented as mean (SD).

Vitamin D modulates muscular strength and power output. Herein, after 28-d of supplemental vitamin D at 200 or 4000 IU/d, and compared to those in the placebo group as well, peak (Figure 4A) or average (Figure 4B) isometric forces were not significantly different. Likewise, peak (Figure 5A) and average (Figure 5B) power outputs were not significantly different between supplemental groups. However, there was a significant main effect of time on peak (*P *< 0.05; Figure 5A) and average (*P *< 0.05; Figure 5B) power outputs, thereby indicating a learning effect.

### Correlations

Although supplemental vitamin D did not significantly change isometric forces, peak (*r *= 0.27, *P *< 0.05) and average (*r *= 0.29, *P *< 0.05) isometric forces correlated with serum 25(OH)D concentrations.

## Discussion

Vitamin D regulates inflammatory cytokines and skeletal muscle function, especially in isolated immune cells and various pathophysiological conditions in humans. In the present investigation, we extend those previous reports by providing several original and impactful results obtained from healthy adults during the winter. First, we provide new data identifying serum 25(OH)D and IL-5 concentration decreases. Secondly, supplemental vitamin D at 200 IU/d prevented serum 25(OH)D and IL-5 concentration decreases. Third, supplemental vitamin D at 4000 IU/d induced an immediate increase in serum 25(OH)D concentrations without modulating circulating IL-5. Finally, despite serum 25(OH)D concentrations correlating with muscle strength, supplemental vitamin D did not moderate muscular strength alterations. These unique data indicate that subtle fluctuations in serum 25(OH)D concentrations modulate a T_H2_-type cytokine, and that muscular strength does not parallel the rapid serum 25(OH)D increase induced by a high-daily dose of supplemental vitamin D in young, reportedly active and healthy adults.

As expected, serum 25 (OH) D concentrations responded disparately to different doses of supplemental vitamin D (Figures 1 and 2). Compared to serum 25(OH)D concentrations prior to supplementation, we observed a 15% increase after 7-d, which progressively inflated by ~50% after 28-d of supplemental vitamin D at 4000 IU/d. Thus, in subjects with mean serum 25(OH)D concentrations in the low-30 ng/ml range prior to supplementation, 4000 IU/d of supplemental vitamin D induced a rapid and continuous increase in serum 25(OH)D concentrations during the winter for 28-d. Conversely, in the 200 IU group, serum 25(OH)D concentrations did not change, while serum 25(OH)D concentrations decreased in the placebo group (Figure 2; 21-d and 28-d). These are important findings because they establish consistency with previous studies that identify a decrease in serum 25(OH)D concentrations during the winter [1,38]. Moreover, our results indicate that at our geographical location, 200 IU/d of supplemental vitamin D plus daily dietary intake of vitamin D is adequate to maintain serum 25(OH)D concentrations and vitamin D sufficiency during the winter, at least in adult non-smokers and during 28-d. However, our data obtained at 200 IU/d of supplemental vitamin D dispute previous findings. Specifically, Barnes et al. [38] recently demonstrated that 200 IU of daily vitamin D supplementation was ineffective at maintaining serum 25(OH)D concentrations during winter [38]. The more Northern geographical latitudes (51° and 55°N) and plausibly the longer supplementation duration (22 weeks) in the study by Barnes et al. [38] could be contributing factors for the discrepancy in maintaining serum 25(OH)D concentration between studies that were conducted at the same reported supplemental dose of vitamin D. This interpretation would further suggest the dose of supplemental vitamin D (plus dietary intake) required to maintain a given serum 25(OH)D concentration increases with increasing Northern latitudes during the winter. Another explanation for the discrepancy between Barnes et al. [38] and those herein could be differences in dietary vitamin D intake. Unfortunately, neither Barnes et al. [38] nor do we report vitamin D intake from dietary sources. Future studies are encouraged include dietary sources (and sun exposure) of vitamin D when investigating serum 25(OH)D concentrations at daily doses of supplemental vitamin D that are easily achievable through dietary sources. Finally, it is probable that subjects randomized to the 200 IU group had a lower percentage of body fat compared to those by Barnes et al. [38]. This could potentially lower the physiological needs of vitamin D in the 200 IU group due to less sequestering to adipose tissue. We speculate that the good vitamin D status reflected by our subjects prior to supplementation is indicative of sun exposure during the preceding summer and fall and daily dietary habits that include vitamin D, such as the consumption of oily fish and vitamin D fortified food sources.

A novelty of the present investigation was the IL-5 results obtained with and without supplemental vitamin D (Figure 3C). In the placebo group, there was a transient IL-5 decrease, while in contrast, IL-5 increased in the 200 IU and remained unchanged in the 4000 IU group. Due to the small sample and effect sizes, it is plausible that 200 IU of supplemental vitamin D maintained as opposed to increased IL-5. This premise is logically harmonious with the data obtained in the 4000 IU group, which in sum suggests that supplemental vitamin D maintains serum 25(OH)D and IL-5 concentrations during the winter. However, consistent with our increase in the 200 IU group, Qi et al. [44] demonstrated that vitamin D (i.e., 1,25(OH)D) pre-treatment in rats injected-intraperitoneally with lipopolysaccharide (LPS) increased IL-5 gene expression in the spleen [44]. Similarly, IL-5 production increased in CD4^+ ^cells obtained from mice when incubated in 1,25(OH)D and stimulated with IL-2, phorbol myristate acetate (PMA) and ionomycin [45]. These results indicate that the active metabolite form of vitamin D increases IL-5. However, results vary. In an experimental mice model of pulmonary eosinophilic inflammation, subcutaneous 1,25(OH)D injections abrogated IL-5 production in bronchoalveolar lavage fluid [46]. Furthermore, when stimulated with PMA and ionomycin in the presence of 1,25(OH)D, IL-5 production decreased in T_H0_-cells and remained unchanged in T_H1_- and T_H2_-cells [47]. Regarding no change in IL-5, Yusupov et al. [48] demonstrated that vitamin D (cholecalciferol) supplementation at 2000 IU/d for 3 months was ineffective at altering circulating IL-5 concentrations in young, healthy adults. Thus, these latter studies potentially conflict with our results at 200 IU but are in agreement with those reported herein at 4000 IU. Collectively, these findings highlight that the cytokine-modulating property of vitamin D could be physiologically dependent on the supplemental dose (and/or environmental factors and dietary intake), the immunological challenge (or lack thereof), experimental model (i.e., humans, rodents, or cell), duration of vitamin D treatment, the timing of data collection, and importantly, the form of vitamin D studied.

Regarding the form of vitamin D, most studies have investigated the influence of 1,25(OH)D on inflammatory cytokines *in vitro*, which makes it is difficult to equate or interpret results relative to supplemental D or serum 25(OH)D concentration studies in humans that do not report 1,25(OH)D. Recently, Zhang et al. [49] demonstrated that 15 ng/ml of serum 25(OH)D was ineffective at suppressing LPS-induced cytokine (i.e., IL-6 and TNF-α) production in human monocytes studied *in vitro*. In contrast, 30 ng/ml of serum 25(OH)D significantly inhibited cytokine production and was comparable to that shown at 0.04 ng/ml of 1,25(OH)D [49]. These paramount findings indicate that serum 25(OH)D concentration, and importantly a specific circulating concentration (i.e., ≥ 30 ng/ml), is of physiological relevance regarding the anti-inflammatory property of vitamin D because its availability in the circulation influences the local tissue production of 1,25(OH)D [49].

Vitamin D receptors are located on T-cells and mast cells [50,51], and along with eosinophils, are sources of IL-5 [52]. IL-5 is essential for promoting eosinophil growth, differentiation, survival and activation, and is often expressed with other T_H2_-type cytokines, such as IL-4 and IL-13 [53,54]. GATA-3 is a transcription factor that promotes IL-5 gene expression and promoter-transactivation in T_H2_-cells [48,55]. In CD4^+ ^cells derived from mice, GATA-3 message expression increased when incubated with 1,25(OH)D [56]. In an experimental mouse model of allergic induced-asthma, 1,25(OH)D up-regulated the message expression of GATA-3 [57]. Thus, we speculate that maintaining serum 25(OH)D concentrations is necessary to sustain substrate availability for the conversion to 1,25(OH)D, which subsequently modulates GATA-3 expression and prevents IL-5 decreases. With that said, it is unclear why IL-5 did not increase in the 4000 IU group, unless there was a transient increase (or decrease) that was not detected with the timing of our blood sampling procedures.

Evidence indicates that several types of hyper-eosinophilic syndromes are mediated by IL-5 [58]. In patients with hyper-eosinophic syndrome, an anti-IL-5 monoclonal antibody (i.e., mepolizumab) spared corticosteroid use [59], which is of clinical importance since long-term corticosteroid use is associated with adverse events. Anti-IL-5 treatment also reduced the disposition of extracellular matrix proteins in bronchial biopsies obtained from atopic asthmatic patients [60], suggesting that neutralizing IL-5 minimizes the repair process following airway injury and eosinophilia. However, the increase in IL-5 could be beneficial. In addition to increasing eosinophils, IL-5 enhances immunoglobulin A (IgA) production [61,62], which protects against a variety of viruses and bacterial infections [for review see [55]]. Recently, Halliday et al. [63] found that serum 25(OH)D concentrations inversely correlated with frequency of illness in collegiate athletes. Perhaps, maintaining or increasing IL-5, as observed in our supplemental vitamin D groups (Figure 3C) who also maintained or increased as opposed to decreased serum 25(OH)D concentrations (Figure 2), could be beneficial by inducing IgA and the resistance against infectious challenge, such as the influenza virus [64]. Clearly, this premise warrants additional research.

In contrast to our hypothesis, supplemental vitamin D did not increase muscular strength (Figure 4A and 4B) or power (Figure 5A and 5B) despite serum 25(OH)D concentrations increasing by ~50% in the 4000 IU group (Figure 1). This finding extends previous reports [65-67] suggesting that supplemental vitamin D does not improve muscular strength. However, previous reports, and including the results here, are not consistent with the majority of the data. There could be several reasons for the inconsistencies. First, vitamin D insufficiency or deficiency results in muscular weakness [7,8]. Compromised muscle strength correlated with serum 25(OH) concentrations in vitamin D deficient adolescent girls [7,9]. In vitamin D deficient elderly and stroke and osteomalacia patients, supplemental vitamin D improved muscle strength concurrently with serum 25(OH)D concentrations [15,16,18,68]. Furthermore, increasing serum 25(OH)D concentrations from ~9 to 16 ng/ml improved muscle strength and function; further but less pronounced strength and function improvements occurred from ~16 to 38 ng/ml in elderly [11], thereby indicating a diminished return in muscle strength with increasing serum 25(OH)D concentrations. In the present investigation, serum 25(OH)D concentrations were ≈ 32 ng/ml for all subjects prior to supplementing (Figure 1), and therefore, subjects were vitamin D sufficient prior to supplementing. Evidence supporting vitamin D sufficiency is also provided by the PTH and calcium concentrations (Table 2), which were within normal clinical reference ranges prior to and following supplementation and despite 25(OH)D decreases in the placebo group. Based on the existing literature and the data presented here, individuals with vitamin D insufficiency or deficiency could be more prone to muscular strength improvements with increasing serum 25(OH)D concentrations than those who are already vitamin D sufficient [68,69]. This theory would explain why muscle strength did not improve despite an increase in serum 25(OH)D concentrations following vitamin D supplementation at 4000 IU/d in subjects who were already vitamin D sufficient.

Another probable explanation for the inconsistencies in the literature is that there are few prospective, randomized studies investigating the influence of supplemental vitamin D on serum 25(OH)D concentrations and muscular strength. The majority of the evidence identifying the beneficial influence of vitamin D on muscular strength or physical performance is correlative [9,11,12,14,69] or between groups demarcated on circulating 25(OH)D concentrations [13,19]. From these data, authors have concluded that increasing circulating 25(OH)D concentrations improves muscular strength or physical performance. Finally, our study was conducted in young adults (men and women). Studies identifying the positive influence of supplemental vitamin D on muscular-based outcomes have been conducted in elderly [10,15], in patients with diverse diseases or illnesses [16-18] or in experimental animals [70,71]. It is plausible that healthy lifestyles in young adults mask the influence of supplemental vitamin D on muscular strength [72]. Although few arguments exist refuting the influence of vitamin D on muscular strength, future randomized trials incorporating various vitamin D dosing regimens that increase serum 25(OH)D concentrations differentially are required to confirm causation in young adults.

Study limitations include: first, a rather short-intervention phase. Future studies are encouraged to conduct longer durations of supplemental interventions when examining the influence of vitamin D on inflammatory cytokines and muscular strength. Second, the level of physical activity was not stringently examined. Recording activities and activity intensities and volumes performed during the preceding seasons both indoor and outdoor is recommended. Third, this study consisted of thirty subjects total (*n *= 10/group). Future studies investigating the influence of different doses of supplemental vitamin D on inflammatory cytokines and muscle strength are encouraged to include a larger sample size. Fourth, our data collection was limited to weekly blood draws and emerging evidence is suggesting that the cytokine modulating property of vitamin D could be time sensitive [34]. Thus, future studies should consider the temporal cytokine response mediated by vitamin D, especially at higher doses of supplemental vitamin D that increase serum 25(OH)D concentrations rapidly. Next, there was variability across time in the cytokine data. To account for this variability statistically, we performed a rank transformation to achieve normality and equal variance. Finally, serum 25(OH)D, inflammatory cytokines and muscular strength might respond differently in diverse populations or conditions, such as in obese, smokers, elderly or critically ill. Thus, extrapolating the present findings to other populations is not recommended.

In summary, low and high doses of supplemental vitamin D prevented serum 25(OH)D and IL-5 decreases in vitamin D sufficient adults during the winter. These impactful findings defy current belief by suggesting that a low dose of supplemental vitamin D (plus dietary intake) that is easily attainable through dietary sources maintains serum 25(OH)D and IL-5 concentrations.

Additionally, despite concentrations correlating with muscle strength, our shocking data reveal that muscular strength does not parallel the increase in serum 25(OH)D concentrations induced by supplemental vitamin D at 4000 IU/d. We conclude that maintaining serum 25(OH)D concentration during the winter prevents a T_H2_-type cytokine decrease, which could be influential in protecting against viral and bacterial infections; and apparently in reportedly healthy and vitamin D sufficient adults, that a further increase in serum 25(OH)D mediated by a high dose of supplemental vitamin D does not improve muscular strength.

## Abbreviations

1α-OHase: 1α-hydroxylase; 1,25(OH)D: 1,25-dihydroxyvitamin D; 25(OH)D: 25-hydroxyvitamin D; eGFR: Estimated glomerular filtration rate; IL: Interleukin; IFN: Interferon; PTH: Parathyroid hormone; T_H1_: T helper 1; T_H2_: T helper 2.

## Competing interests

The authors declare that they have no competing interests.

## Authors' contributions

TB contributed to the study conception and experimental design, acquisition of data, analysis and interpretation of data, and first draft of the manuscript. TBM carried out the cytokine analysis, BMD, EDS and AD carried out the vitamin D metabolite measurements, VTH performed the single-leg strength analyses. HRH, CRK, BMD and LKW made significant contributions to the critical revision and intellectual content of this manuscript. All authors approved the final manuscript version.
